# Clinical application of 3D Slicer combined with Sina/MosoCam multimodal system in preoperative planning of brain lesions surgery

**DOI:** 10.1038/s41598-022-22549-7

**Published:** 2022-11-10

**Authors:** Long Zhou, Wenju Wang, Hangyu Wei, Ping Song, Zhiyang Li, Li Cheng, Pan Lei, Qianxue Chen, Zaiming Liu, Hui Ye, Qiang Cai

**Affiliations:** 1 Department of Neurosurgery, Renmin Hospital of Wuhan University, No. 238, Jiefang Road, Wuchang District, Wuhan, 430060 Hubei China; 2 Department of Critical Care Medicine, Eastern Campus, Renmin Hospital of Wuhan University, Wuhan, Hubei China

**Keywords:** Diseases of the nervous system, Neurology, Oncology

## Abstract

To explore the clinical advantages of 3D Slicer combined with Sina/MosoCam multimodal system in preoperative planning of brain lesions surgery. By collecting the data of brain lesions patients undergoing craniotomy under the preoperative positioning of 3D Slicer combined Sina/MosoCam multimodal system in the people's Hospital of Wuhan University from January 2021 to October 2021, the preoperative planning of patients was introduced, and the size of surgical bone window, operation time, preoperative and postoperative neurological dysfunction were counted. We collected the case data of 35 patients who were reconstructed by 3D Slicer and located by Sina/MosoCam projection. There were 14 cases of malignant tumors (7 cases of glioma, 2 cases of diffuse large B-cell lymphoma, 5 cases of metastatic cancer) and 21 cases of benign tumors (17 cases of meningioma, 1 case of central neurocytoma, 2 cases of cavernous hemangioma and 1 case of arachnoid cyst). All 35 patients were located accurately before operation, the lesions were found quickly during operation, and the postoperative imaging data confirmed that the lesions were removed completely, of which 28 cases (80%) had significantly improved neurological symptoms one month after operation. 3D Slicer combined with Sina/MosoCam multimodal system has many advantages, such as simple and easy to learn, convenient operation, accurate positioning and free. It is considered to be a new technology that is practical, reliable, convenient for diagnosis and preoperative planning. It is suitable for popularization and use in neurosurgery and other operating rooms of all medical institutions.

## Introduction

With the improvement of people's living standards, brain lesions have gradually developed into the second largest intracranial disease threatening human health after stroke, and craniotomy is the most direct and effective method to treat brain lesions. The main goal of brain lesions surgery is to maximize lesions resection, reduce complications and do not cause new neurological dysfunction. Therefore, accurate preoperative planning is very important. 3D Slicer is a free open-source medical image processing software, which can run smoothly on personal computers and is compatible with multiple systems at the same time; It has been used in the treatment of various diseases, including intracerebral hemorrhage, intracranial aneurysm, brain tumor, trigeminal neuralgia and other neurological diseases^1,2^. 3D Slicer can clearly realize the relationship between the lesion and the nerves and blood vessels, including adjacent, inclusion and invasion, through three-dimensional reconstruction of brain tumors^3^. Sina/MosoCam is an application designed for Android/Apple smartphones. This approach uses a double-exposure technique to superimpose a static image of the 3D Slicer-based surgical plan over a live video image of the patient. By manually positioning the phone so that the video matches the virtual camera view of the patient , the surgical plan can be transferred to the patient's skin by surgical team^1,4^. In this study, the virtual three-dimensional image reconstructed by Sina/MosoCam projection 3D Slicer can accurately locate the body surface position of brain lesions, significantly improve the operation efficiency and safety, reduce the operation trauma and neurological side injury, and reduce the incidence of various complications.

## Clinical data

In this study, the data of patients with brain tumors in the Department of Neurosurgery of the Renmin Hospital of Wuhan University from January 2021 to October 2021 were collected. The inclusion criteria were patients who were reconstructed by 3D Slicer (the version 4.10.2. link: https://slicer.org) and located by Sina/MosoCam projection (Video 1), including meningiomas, gliomas, intracranial metastases, cavernous hemangiomas and so on. Collect the data of preoperative MRI+ enhancement or brain CTA and brain thin-layer CT. The data format is digital imaging and communications in medicine (DICOM), preoperative 3D Slicer reconstruction images, planning of surgical approach, projection positioning and surgical incision design. A total of 35 patients with complete data were collected, including 13 males and 22 females, aged 31–76 years, including 14 malignant tumors (7 gliomas, 2 diffuse large B-cell lymphoma and 5 metastatic cancers), 21 benign tumors (17 meningiomas, 1 central neurocytoma, 2 cavernous hemangiomas and 1 arachnoid cyst). All 35 patients had accurate preoperative localization and quickly found the tumor during the operation. The postoperative imaging data confirmed that the tumor was removed cleanly, and the success rate of the operation was 100%. Among them, 28 patients had significantly improved neurological symptoms one month after the operation, and the symptom improvement rate was 80%. The detailed clinical information of 35 patients is shown in Table 1.

**Table 1:** The detailed clinical information.

Number	Gender	Age	Histology		Position	Space occupying volume (cm^3^)	Operation time(h)	Bone window area (cm^2^)	Nervous system signs	
			Pathology	WHO classification					Preoperative	1 month after operation
1	M	55	Anaplastic astrocytoma	III	Right frontal lobe	3 × 4.5 × 4.6	3.80	5.5 × 5.5	Convulsions and disturbance of consciousness	Normal
2	M	71	Diffuse large B-cell lymphoma	–	Left basal ganglia	4 × 4 × 3	4.18	5 × 5	Grade 0 of right upper limb and grade III of right lower limb	Right upper limb grade 0 and Right lower limb grade III
3	F	67	Brain metastasis of lung cancer	–	Right lateral ventricular triangle	2.4 × 3.6 × 2.1	5.63	6 × 8	Dizziness and left lower limb muscle strength grade III	Left lower limb muscle strength grade IV
4	F	37	Central neurocytoma	II	Right ventricle	3.5 × 3.3 × 3.5	3.62	3 × 3.5	Headache and nausea and vomiting	Hydrocephalus and ventriculoperitoneal shunt
5	F	67	Meningioma	I	Left precentral gyrus	1 × 1.5 × 1	2.20	3 × 3	Dizziness, unstable standing and blurred vision	Blurred vision
6	F	76	Meningioma	I	Left frontal part	3.8 × 4.6 × 2.3	4.32	7 × 8	Normal	Normal
7	M	68	Meningioma	I	Beside the left frontal sickle	2.3 × 2.8 × 3.0	2.20	3.5 × 4	Intermittent headache and anosmia	Anosmia
8	F	74	Meningioma	III	Right parietal lobe	4.7 × 3.1 × 3.6	3.30	5 × 6	Dizziness and unsteady walking	Normal
9	M	36	Glioblastoma	IV	Left frontal parietal lobe	3.0 × 2.9 × 3.0	5.05	5 × 5	Right limb grade II	Right limb grade IV
10	M	40	Meningioma	I	Left frontal lobe	5 × 6 × 5	2.67	5.5 × 5.5	Dizziness and right limb grade III	Normal
11	M	51	Glioblastoma	IV	Right frontal lobe	3.9 × 3.2 × 3	3.40	5.5 × 6	Convulsion, grade 0 of left upper limb and grade IV of lower limb	Left upper limb grade II and lower limb grade IV
12	F	53	Meningioma	I	Left temporal lobe	3.6 × 4.3 × 4.5	3.48	3.5 × 4.5	Right limb grade IV with pain	Normal
13	M	52	Oligodendroglioma	II	Right frontal lobe	3.1 × 2.7 × 2	2.07	4 × 4.5	Convulsions and disturbance of consciousness	Normal
14	F	46	Cavernous hemangioma	–	Brainstem	1 × 1.8 × 1	4.57	4 × 5.5	Blepharoptosis and visual ghosting	Blepharoptosis, blurred vision, left muscle strength grade III
15	F	60	Diffuse large B-cell lymphoma	–	Right basal ganglia	3 × 2 × 2	3.92	3.5 × 3.5	Hypomnesia	Left limb grade IV
16	F	59	Meningioma	I	Left parietal lobe	1.5 × 1.1 × 1.2	2.83	3.5 × 4	Intermittent headache	Normal
17	M	31	Arachnoid cyst	–	Right frontal lobe	4.4 × 3.9 × 4.3	4.38	4.5 × 4.5	Normal	Normal
18	F	44	Meningioma	I	Right frontal lobe	1.1 × 1.3 × 1.4	1.87	3.5 × 3.5	Normal	Normal
19	M	57	Meningioma	I	Beside the left parietal sickle	1.9 × 1.5 × 1.4	2.35	2 × 3.5	Convulsions and disturbance of consciousness	Normal
20	F	59	hemangiopericytoma	–	Right lateral ventricular triangle	3.5 × 5.2 × 4.1	4.62	7 × 7	Left lower limb IV	Normal
21	F	42	Meningioma	I	Beside the left parietal sickle	2 × 2.5 × 2	5.27	4 × 5	Dizziness and vomiting, right limb grade IV	Normal
22	F	74	Glioblastoma	IV	Right temporal lobe	5 × 4 × 3	6.02	6 × 8	Mental disorders and hypomnesia	Stupor
23	F	63	Meningioma	I	Left anterior skull base	3.8 × 3.3 × 4 and 2 × 1 × 1	5.25	5 × 6 and 3 × 3	Headache	Anosmia
24	F	38	Meningioma	II	Beside the left parietal sickle	5.8 × 6 × 6	5.83	7 × 7	Headache	Right limb grade IV
25	F	46	Brain metastases from breast cancer	–	Right frontal parietal lobe and cerebellum	3.1 × 2.5 × 3 and 1 × 1 × 1.5	4.10	3.5 × 3.5 and 2.5 × 3	Dizziness, grade IV and uncoordinated left limb	Left limb grade IV and disharmony
26	F	70	Meningioma	I	Left parafrontal parietal falx	5 × 6 × 7	5.50	6 × 7	Convulsions	The right upper limb is grade IV and the lower limb is grade II
27	M	69	Brain metastasis of lung cancer	–	Left temporal lobe and right temporal lobe	2.1 × 2.2 × 2 and 1 × 0.5 × 1	6.48	3 × 4 and 3 × 3	Headache and dizziness	Left limb grade II and right limb grade IV
28	F	59	Meningioma	I	Anterior skull base	4 × 5 × 4	5.82	5 × 8	Olfactory visual impairment	Improved vision and loss of smell
29	F	65	Meningioma	I	Left parietal lobe	3.5 × 3.4 × 3.8	5.10	5 × 6	Right limb grade IV	Normal
30	F	57	Meningioma	I	Left frontal lobe	2 × 1.5 × 2	2.08	4.5 × 4.5	Headache	Normal
31	F	62	Glioblastoma	IV	Right frontotemporal lobe	5.9 × 2.9 × 5.0	3.98	5.5 × 6	Dizziness	A few mental disorders and speech disorders
32	M	63	Cavernous hemangioma	–	Right septum pellucidum	1.5*1.0*0.5	3.13	4 × 4	Left upper limb grade III	Left upper limb grade IV
33	M	61	Brain metastasis of lung cancer	–	Left parietal lobe	1.8 × 2.1 × 2.2	3.63	6 × 6	Headache with paresthesia of both upper limbs	Normal
34	M	52	Brain metastasis of lung cancer	–	Right thalamus	1.9 × 2.2 × 1.5	4.55	5 × 5	Left limb grade III	Left limb grade IV
35	F	51	Glioblastoma	IV	Left temporal parietal occipital lobe	7.8 × 5.3 × 4.8	3.40	6 × 7	Headache with hypomnesis	Normal

### Ethical approval

All procedures performed in studies involving human participants were in accordance with the ethical standards of the institutional and/or national research committee and with the 1964 Helsinki declaration and its later amendments or comparable ethical standards. And it has been approved by the Ethics Committee of Clinical Research, Renmin Hospital of Wuhan University (WDRY2022-KS003). It has been confirmed that we have obtained the informed consent of the patients and their families for their images to be published in an online, fully open access journal.

### Informed consent

Informed consent was obtained from all individual participants included in the study.

### Typical cases data

**Figure 1:**
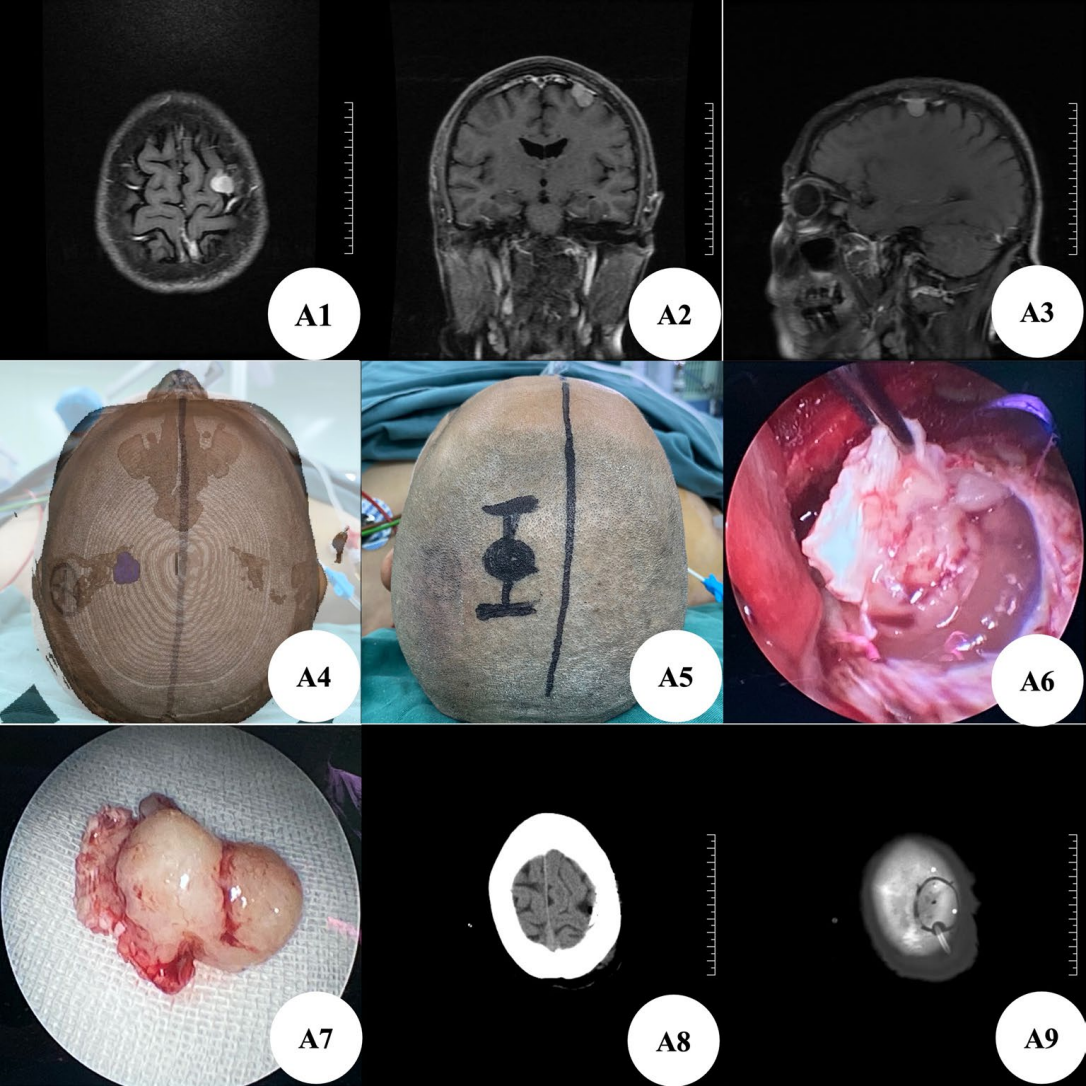
(**A1–A3**) Brain MRI enhancement showed left frontal meningioma; (**A4**) use the Apple mobile phone APP MosoCam for projection positioning; (**A5**) the surgical incision was designed according to the projection location; (**A6**) intraoperative neuroendoscopic brain tumor; (**A7**) total tumor specimens; (**A8**) postoperative brain CT showed that the tumor was resected cleanly without delayed bleeding; (**A9**) brain CT showed the size of bone window.

Patient A, female, was hospitalized for 1 year due to severed head pain. Brain MRI + enhancement showed left frontal lobe meningioma, with a size of about 1.5 cm × 1.1 cm × 1.2 cm (Fig. 1A1–A3). At the time of admission, the patient had no positive manifestations of nervous system except intermittent head pain. According to the preoperative thin-layer CT scanning data of brain, we used 3D Slicer software for three-dimensional reconstruction to plan the most appropriate surgical approach (Fig. 1A4). Before operation, MosoCam projection APP for Apple mobile phone was used to project the scalp surface of the tumor, mark, and scribe, and design a 4 cm straight incision (Fig. 1A5). After cutting the scalp according to the marked surgical incision, mill a bone flap with a diameter of about 3 cm with a milling cutter, and the lesion dura under the bone flap can be seen immediately. Carefully cut the dura along the edge to show the tumor (Fig. 1A6), accurately locate and completely remove the tumor (Fig. 1A7). The post-operative brain CT showed that the tumor was resected clean (Fig. 1A8,A9), and the postoperative pathological examination showed that the meningioma was fibrous and WHO grade I. He recovered and was discharged on the 10th day after operation without any neurological symptoms.

**Figure 2:**
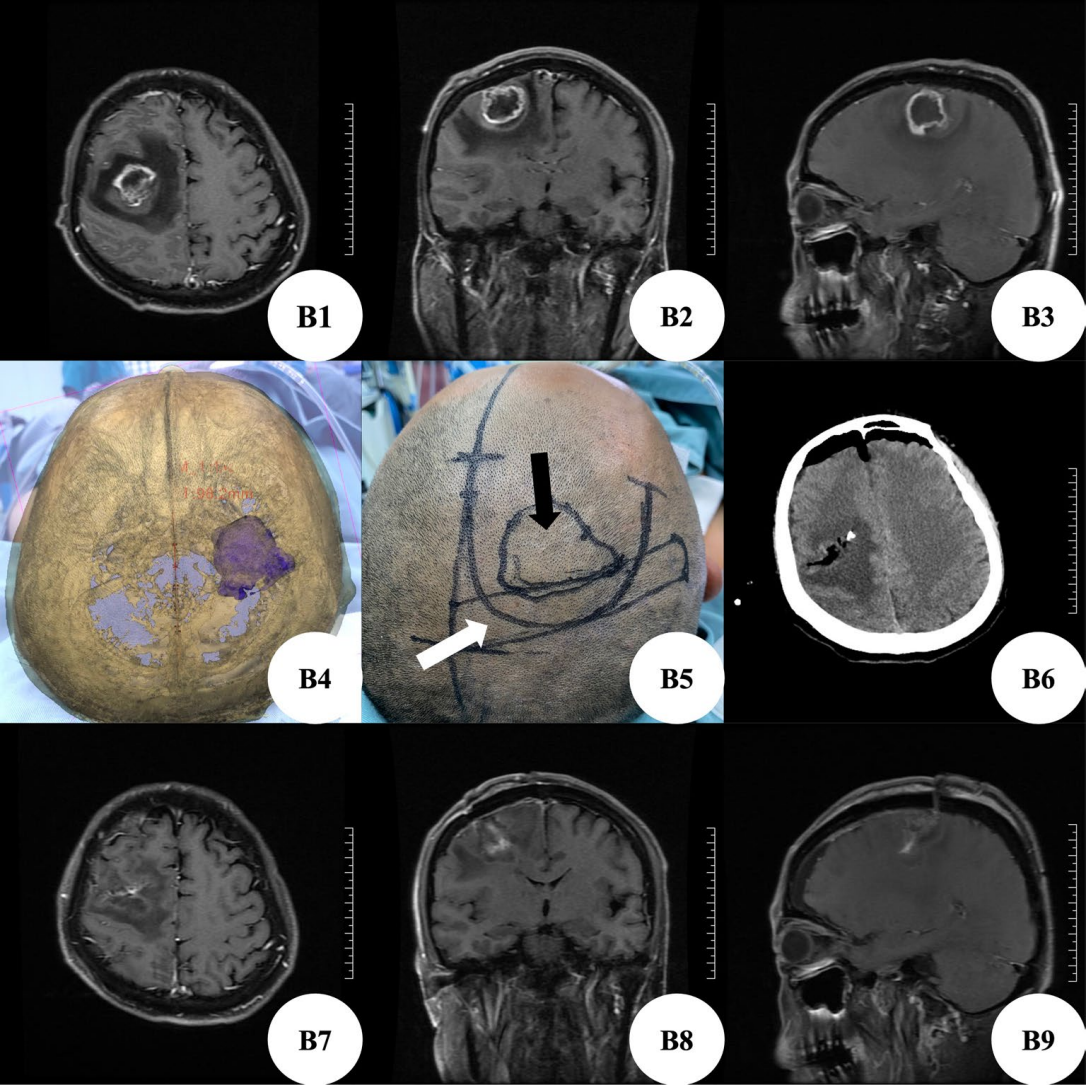
(**B1–B3**) Enhanced brain MRI showed space occupying lesions in the right frontal lobe; (**B4**) use the Apple mobile phone APP MosoCam for projection positioning; (**B5**) the surgical incision was designed according to the projection location. The black arrow was the body surface projection of the tumor, and the white arrow was the body surface projection of the central anterior gyrus; (**B6**) postoperative brain CT showed that the tumor was resected cleanly without delayed bleeding; (**B7–B9**) on the 7th day after operation, brain MRI enhanced examination showed that there was no residual tumor.

Patient B, male, was hospitalized because of sudden convulsion for 20 days, left limb fatigue for 3 days and aggravated for 1 day. Brain MRI+ enhancement showed space occupying lesions in the right frontal lobe, with a size of about 3.9 cm × 3.2 cm × 3 cm, glioma may occur, and the midline deviates to the left (Fig. 2B1–B3). At admission, the patient was conscious, the muscle strength of the left upper limb was grade 0, the muscle strength of the left lower limb was grade IV, the muscle strength of the right limb was normal, there were no other positive signs of nervous system, and there were no convulsions again. According to the preoperative brain MRI scanning data, we used 3D Slicer software for three-dimensional reconstruction to plan the most appropriate surgical approach (Fig. 2B4). Before operation, MosoCam projection APP for Apple mobile phone was used to project the scalp surface of the tumor, mark and scribe, and design a "U" shaped surgical incision (Fig. 2B5). After cutting the scalp according to the marked surgical incision, the milling cutter milled a bone flap with a diameter of about 5 cm. After exploration, it was found that the anterior area of the central anterior gyrus of the right frontal lobe was swollen obviously, gray-white, rich in blood supply, without obvious boundary and capsule, and tough in texture. The tumor originated from brain tissue, invaded the central anterior gyrus backward, separated and resected along the edema area of the tumor boundary, with accurate positioning, complete resection of the tumor. Postoperative brain CT showed that the tumor was resected clean and there was no delayed bleeding in the operation area (Fig. 2B6); postoperative pathological examination showed glioblastoma, IDH mutant, WHO grade IV. On the 7th day after operation, the brain MRI+ enhancement examination showed that there was no residual tumor (Fig. 2B7–B9). On the 11th day after operation, he recovered and was discharged from the hospital with clear mind, grade II muscle strength of the left upper limb, grade IV muscle strength of the left lower limb, and normal muscle strength of the right limb.

**Figure 3:**
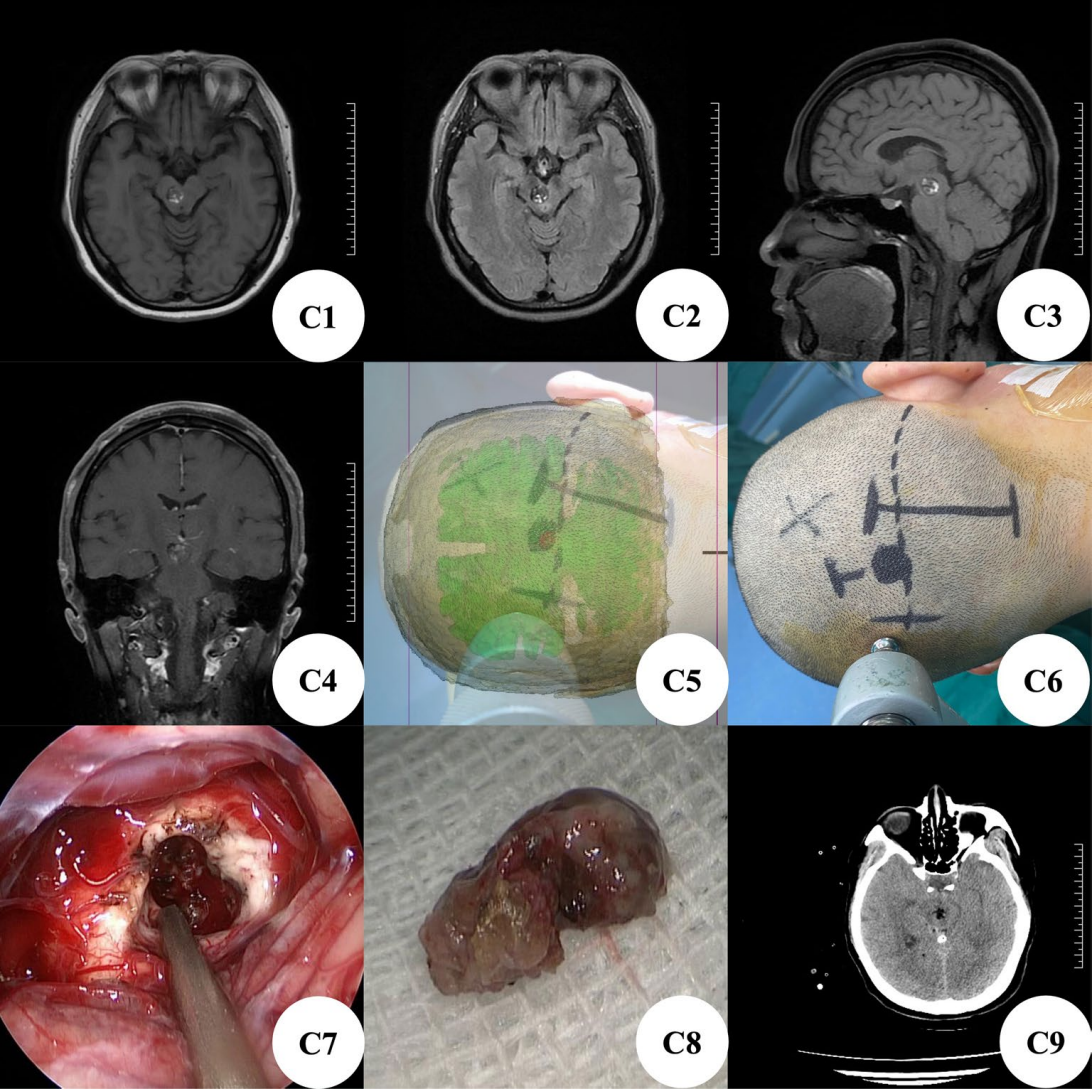
(**C1–C4**) MRI examination of the brain showed space occupying lesions on the right side of the midbrain and cavernous hemangioma; (**C5**) use the Apple mobile phone APP MosoCam to project the scalp surface of the tumor; (**C6**) the surgical incision was designed according to the projection position; (**C7**) cavernous hemangioma was found by neuroendoscopy during operation; (**C8**) complete resection specimens of cavernous hemangioma; (**C9**) postoperative brain CT showed that the cavernous hemangioma was removed completely and there was no delayed bleeding in the operation area.

Patient C, female, was hospitalized because of right eyelid ptosis with visual ghosting for 20 days. Brain MRI+ enhancement showed a cavernous hemangioma on the right side of the midbrain, with peritumoral venous malformation, with a size of about 1 cm × 1.8 cm × 1 cm (Fig. 3C1–C4). At admission, the patient was clearly conscious, the bilateral pupils were unequal in size, the left pupil was about 2 mm in diameter, sensitive to direct light reflection, and the right pupil was about 3.5 mm in diameter, dull to direct light reflection, visual ghosting, limited upward, downward and inward movement of the right eye, showing an external booth, the muscle strength and tension of limbs were normal, and there were no other positive signs of nervous system. According to the preoperative CTA thin-layer scanning data of brain, we used 3D Slicer software for three-dimensional reconstruction to plan the most appropriate surgical approach (Fig. 3C5). Before operation, MosoCam projection APP for Apple mobile phone was used to project the scalp surface of the tumor, mark and scribe, and design a straight incision of about 8 cm (Fig. 3C6). After cutting the scalp according to the marked surgical incision, expose the occipital bone and transverse sinus area, mill a bone flap with a diameter of about 4 cm with a milling cutter, dissect the supratentorial cerebellum and annular cistern area, enter the midbrain area, carefully explore, and enter the midbrain along the lateral sulcus of the midbrain. It can be seen that the lesion is located in the right midbrain, with tough texture, rich blood supply, small blood supply arteries and drainage veins, bleeding and clear boundary, there is no obvious capsule, the surrounding brain tissue has yellowing, the lesion can be seen under neuroendoscope (Fig. 3C7), the location is accurate, and the space occupying lesion is completely removed (Fig. 3C8). Postoperative brain CT reexamination showed that the midbrain mass was removed completely and there was no delayed hemorrhage in the brain stem (Fig. 3C9), postoperative pathological examination showed cavernous hemangioma. On the 17th day after operation, the brain CT showed that the brain stem lesions were removed completely, there was no delayed hemorrhage, and the brain stem edema subsided significantly. On the 18th day after operation, he recovered and was discharged from the hospital with clear mind. The diameter of the left pupil was about 2 mm, the light reflex was sensitive, the diameter of the right pupil was about 3 mm, the light reflex disappeared, the right eyelid was still drooping, the vision was still blurred, the muscle strength of the left limb was grade III-IV, and the muscle strength of the right limb was normal.

**Figure 4:**
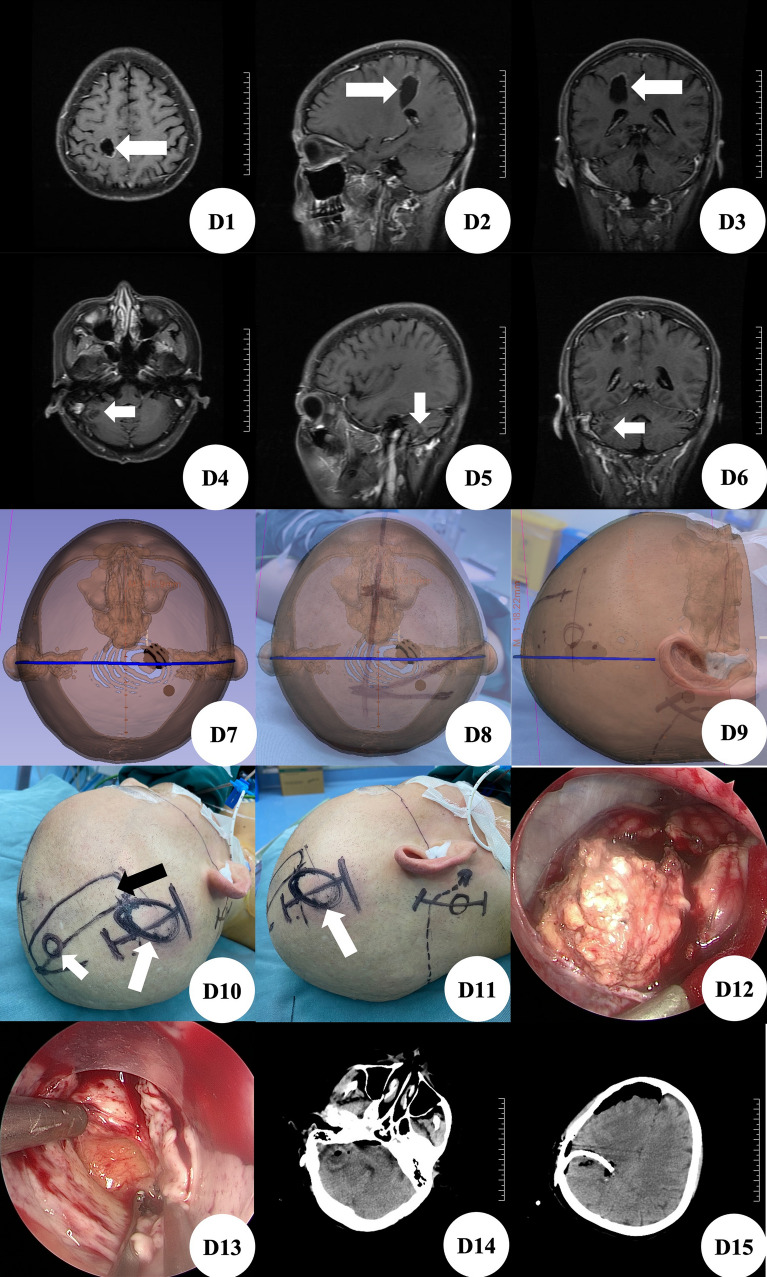
(**D1–D3**) Brain MRI enhancement showed cystic space occupying lesions in the right frontal parietal lobe, indicated by the white arrow; (**D4–D6**) brain MRI enhancement showed solid space occupying lesions in the right cerebellum, indicated by the white arrow; (**D7**) 3D Slicer was used to reconstruct the space occupying image of the right frontal parietal lobe; (**D8,D9**) use the Apple mobile phone APP MosoCam to show the positive and lateral projection of the right top space occupation; (**D10,D11**) according to the tumor location located by MosoCam surgical software and the designed surgical incision, the black arrow is the body surface projection of the central anterior gyrus, and the white arrow is the frontal and lateral projection of the right frontoparietal lobe respectively; (**D12**) intraoperative neuroendoscopic images of right cerebellar mass; (**D13**) intraoperative neuroendoscopic images of right parietal frontal space occupation; (**D14,D15**) postoperative brain CT showed that the tumor was resected cleanly without delayed bleeding.

Patients D, female, was hospitalized because of left breast cancer 10 years after operation, dizziness for 3 months aggravated with left limb weakness for 2 months. Brain MRI+ enhancement showed multiple space occupying lesions in bilateral frontal parietal lobe, left occipital lobe and right cerebellum. The larger one was located in the right frontal parietal lobe, with a size of about 3.1 cm × 2.5 cm × 3 cm, and right cerebellum, with a size of about 1 cm × 1 cm × 1.5 cm in size (Fig. 4D1–D6). At admission, the patient was conscious, the finger nose test of the left upper limb was positive, the heel knee tibia test of the left lower limb was positive, and the right side was normal, the muscle strength of the left limb was grade IV, and the muscle strength of the right limb was normal. According to the preoperative thin-layer CT scanning data of brain, we used 3D Slicer software for three-dimensional reconstruction (Fig. 4D7) to plan the most appropriate surgical approach (Fig. 4D8,D9). Before operation, MosoCam projection APP for Apple mobile phone was used to project the scalp surface of the tumor, mark and scribe, and design the linear skin incision in the hairline behind the right ear and the linear skin incision at the top of the right temporal (Fig. 4D10,D11). Firstly, the scalp was cut according to the surgical incision marked behind the right ear, mill the bone window with a diameter of about 3 cm with a milling cutter, fully expose the transverse sinus and partially expose the sigmoid sinus, carefully cut the dura mater, dissect the occipital cistern, explore and see that the lesion is located in the right cerebellar parenchyma, cut the cortex and enter the lesion, see that the texture of the lesion is tough, the blood supply is general, the boundary is clear, there is no obvious capsule, and there is edema around, After separating the tumor along the edematous tissue under neuroendoscopy, the tumor was removed with accurate tumor localization(Fig. 4D12) and complete resection of tumor. Then the scalp was cut according to the surgical incision marked on the top of the right frontal lobe, the bone window with a diameter of about 3 cm was milled with a milling cutter, and a transparent neuroendoscopic tube sheath was implanted after cortical fistula. The exploration showed that the lesion was located at the junction between the lower part of the central anterior gyrus and the inferior parietal lobule of the right frontal lobe. It was a cystic lesion, the cystic fluid was yellow, cholesterol like, the boundary was still clear, the texture was soft, and the blood supply was general, the tumor was resected after separating the lesion along the boundary, with accurate tumor localization (Fig. 4D13) and complete resection of tumor. The postoperative brain CT showed that the tumors in the right frontal lobe and right cerebellum were removed completely, and there was no obvious bleeding in the operation area (Fig. 4D14,D15). After surgery, two specimens showed infiltration of cancer cells, which was in line with metastasis of breast cancer. On the 15th day after operation, he recovered and was discharged from the hospital with clear mind. The finger nose test of the left upper limb was positive, the heel knee tibia test of the left lower limb was positive, and the right side was normal, the muscle strength of the left limb was grade III, and the muscle strength of the right limb was normal.

**Figure 5:**
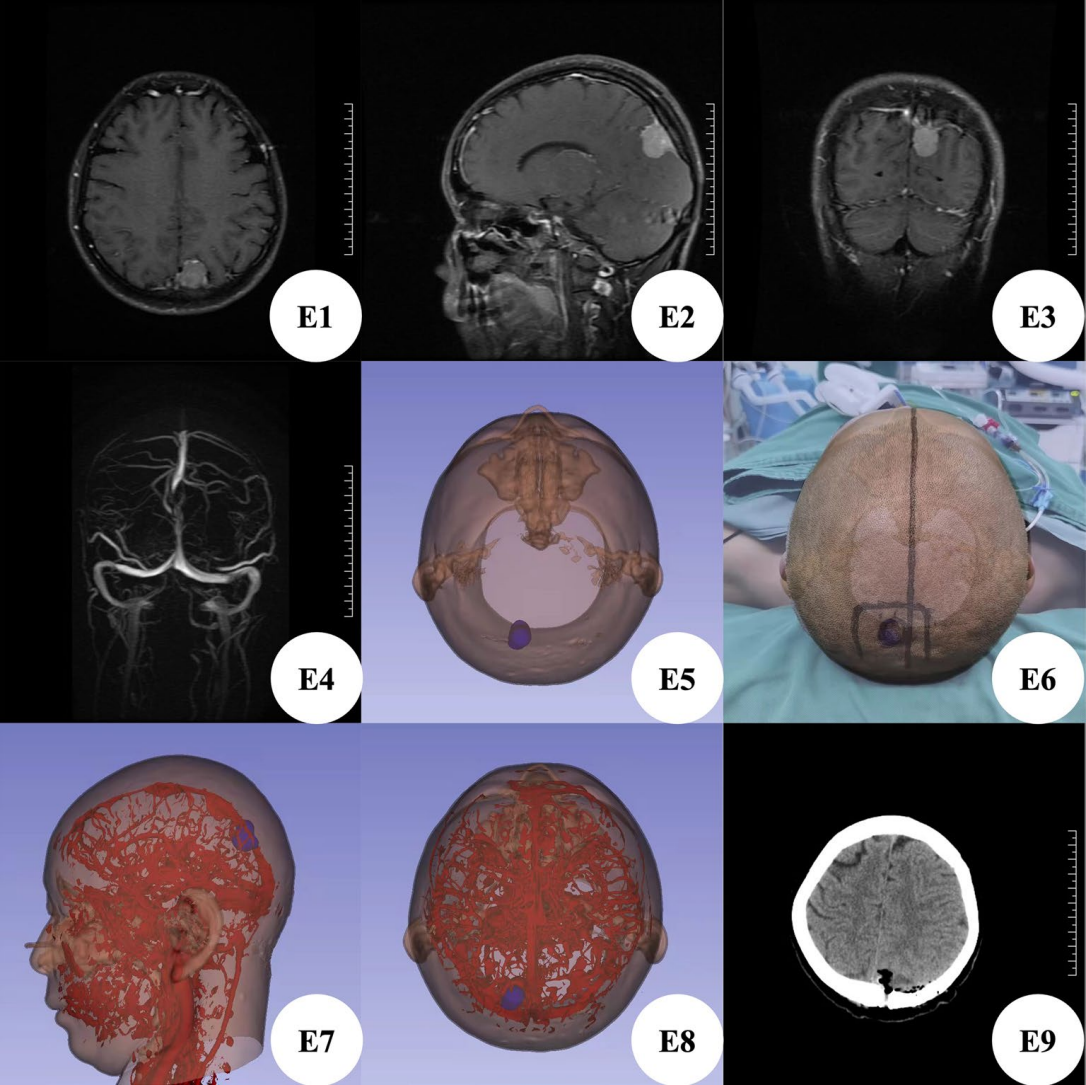
(**E1–E3**) Brain MRI enhancement showed left parietal meningioma; (**E4**) brain MRV showed that the posterior part of intracranial superior sagittal sinus became narrower; (**E5**) 3D Slicer was used to reconstruct the left parietal meningioma image; (**E6**) the Apple mobile phone APP MosoCam was used to design the left parietal space occupying projection and surgical incision; (**E7,E8**) 3D Slicer was used to reconstruct the image of the relationship between intracranial vessels and left parietal meningioma; (**E9**) postoperative brain CT showed that the tumor was resected cleanly without delayed bleeding.

Patient E, female, was hospitalized due to dizziness and vomiting for 14 h. Brain MRI + enhanced examination showed a space occupying lesion in the left parietal lobe, with a size of about 2 cm × 2.5 cm × 2 cm, and a possible meningioma (Fig. 5E1–E3), Brain MRV showed that the posterior part of the intracranial superior sagittal sinus narrowed (Fig. 5E4), which was caused by the compression of the left parietal lobe. At admission, the patient was conscious, but with dizziness, vomiting and weakness of the right limb. According to the preoperative CTA thin-layer scanning data of brain, we used 3D Slicer software for three-dimensional reconstruction (Fig. 5E5), planned the most appropriate surgical approach, and reconstructed the positional relationship between blood vessels and tumors (Fig. 5E7,E8). Before operation, MosoCam mobile phone projection software was used to project the scalp surface of the tumor (Fig. 5E6) and mark and scribe. Considering that the tumor squeezed the sagittal sinus, we did not design a simple straight incision, but chose the left top inverted "U" skin incision across the midline. After marking, the patient took the prone position for operation. After cutting the scalp according to the marked surgical incision, mill a bone flap with a diameter of about 4 cm × 4 cm with a milling cutter. Immediately, the tumor erodes the inner plate of the skull and destroys the dura mater. Carefully cut the dura mater along the edge and found that he tumor base is located on the superior sagittal sinus and falx cerebri. The tumor adheres closely to the bridging vein draining into the superior sagittal sinus. Carefully separate the adhesion between the tumor and the bridging vein, and cut off the tumor base first, block resection of tumor, complete protection of blood vessels and sagittal sinus, accurate tumor location and complete resection of tumor. The post-operative brain CT showed that the tumor was resected cleanly (Fig. 5E9), and the postoperative pathological examination showed that the meningioma was of mixed type and WHO grade I. He recovered and was discharged on the 15th day after operation without any neurological symptoms.

## Discussion

The long-term survival and high-quality life of patients with brain tumors depend on the scope of tumor resection and the protection of functional white matter fibers. Therefore, neurosurgery needs accurate structural positioning. For this reason, the application of preoperative reasonable planning system is an important embodiment of modern medical technology in brain surgery. With the rapid development of computer technology and radiology, surgeons can plan and simulate the most appropriate operation mode before operation. As a new imaging tool, multimodal fusion imaging technology such as 3D Slicer has been widely used in the field of neurosurgery in recent years^1^. At present, 3D Slicer software is developing at an amazing speed in the field of neurosurgery. It is characterized by free and open source, simple operation, low requirements for computer configuration, functional expansion and continuous optimization and upgrading. With the rapid development of medical imaging, neurosurgeons can fuse the data in DICOM format of patients' thin-layer CT or MRI through 3D Slicer software, so as to obtain the multi-module three-dimensional model of brain tumor. Then the virtual three-dimensional digital model was matched with the body surface markers on the patient's scalp surface by Sina/MosoCam mobile phone projection software^1^, so as to depict the outline of the lesion and assist clinical neurosurgeons to realize the accurate positioning of the preoperative lesion. It not only has the function similar to the neuronavigational system, but also can achieve the accurate positioning of the lesion without the disadvantages of expensive and complex operation of the neuronavigational system.

It is an arduous task for neurosurgeons to design a safe and special surgical method to protect the central nervous system. 3D Slicer combined with Sina/MosoCam software can formulate individualized operation plan before operation, avoid important brain functional areas and large blood vessels adjacent to brain tumors, plan the best operation approach and simulate the design of surgical incision^5^. Our research shows that for meningiomas with superficial location, we can achieve accurate preoperative positioning, change the previous scalp incision method of using "U" shaped or arc-shaped large incision due to inaccurate preoperative positioning, and adopt the direct skin incision to directly reach the tumor, significantly reduce the surgical trauma, accurately locate and avoid unnecessary damage to normal brain tissue and nerve fibers, and shorten the time of postoperative rehabilitation. For tumors located in deep or functional areas, total resection is extremely challenging. It is extremely difficult to completely cut tumors without side injury on the premise of protecting function. However, we reconstructed the tumor through 3D Slicer, understood the relationship with the surrounding structure through software rotation, designed the most suitable new surgical approach, projected the scalp surface of the tumor through Sina/MosoCam mobile phone projection software, accurately located the tumor, successfully completed the total tumor resection and avoided serious complications. Case A is a right frontal meningioma. The location of the tumor was determined through preoperative 3D Slicer reconstruction, the most appropriate surgical approach and scalp positioning point were designed, and the preoperative planning was accurately completed through MosoCam mobile phone software projection. The advantages of this technology were also confirmed during and after operation. Case B is a glioblastoma of the left frontal lobe. From the imaging point of view, the tumor and edema zone affect the important functional structures of the anterior central gyrus. There is hemiplegia of the contralateral limb before operation, and the positioning is inaccurate. However, it causes side injury of the operation, and it is very easy to have permanent hemiplegia of the contralateral limb, affecting the quality of life of the patient after operation. We used 3D Slicer to reconstruct the location of the lesion and understand the positional relationship between the lesion and the anterior central gyrus. Before operation, we used MosoCam mobile phone software for accurate projection, which perfectly avoided the important functional structures such as the anterior central gyrus. After operation, the muscle strength of the contralateral limb of the patient was significantly improved, and it is completely possible to restore normal function through rehabilitation treatment in the later stage. Case C is a cavernous hemangioma of the brain stem. The lesion is deep and located in the important structure of the midbrain. A slight carelessness may cause postoperative hemiplegia, and in severe cases may lead to coma or even death. Before the operation, we used 3D Slicer to reconstruct the location of the lesion and MosoCam mobile phone software for accurate projection. We skillfully avoided important blood vessels, nerves and other key brain tissue structures, completely removed the lesion. The patient had clear consciousness after the operation, and only slight hemiplegia gradually recovered after later rehabilitation treatment. Case D was multiple metastases of breast cancer, with lesions located on the same side. We used 3D Slicer to reconstruct the location of the focus, cleverly designed the surgical incision, and accurately projected it through MosoCam mobile phone software before operation. The supratentorial and infratentorial lesions were removed in one operation. The surgical trauma was small, the patients were more acceptable psychologically, and the postoperative recovery was faster. Case E is a parasagittal meningioma. We used 3D Slicer reconstruction to clarify the relationship between the tumor and the sagittal sinus and found that there were perforating vessels passing through the tumor. The scalp surface of the tumor was projected by Sina mobile phone projection software to accurately locate the tumor. It avoids the side injury caused by inaccurate positioning and the risk of massive bleeding caused by vascular injury during tumor resection. Through our clinical retrospective study, we found that 3D Slicer combined with Sina/MosoCam multimodal system is not only suitable for preoperative planning of intracranial convex lesions, but also suitable for preoperative planning of deep brain lesions. This multimodal preoperative planning system provides a simple, economical and reliable solution for surgical planning and implementation, and there is no need to use the traditional expensive and complex intraoperative neuronavigational system.

3D Slicer combined with Sina/MosoCam multimodal system is widely used in neurosurgery, but it also has shortcomings like other preoperative and intraoperative image guidance systems. Sina/MosoCam projection system allows the preoperative three-dimensional image space to be projected into the surgical space, but the positioning accuracy is affected by the operator's technology^6^ and other clinical factors. Secondly, once the dura mater is opened, the brain will shift before the actual start of surgical treatment. At the beginning of the actual operation, the essence of the lesion may continue to change compared with the preoperative imaging examination. This can be clearly shown from our postoperative brain CT. Almost all patients have obvious brain tissue displacement and postoperative pneumocephalus. Clinical studies show^7^ that brain displacement is mainly caused by cerebrospinal fluid drainage after subarachnoid opening, focal tissue resection, traction of superficial brain tissue in order to remove deep lesions, and administration of drugs affecting brain tissue perfusion during operation. Even if the intraoperative navigation system provides accurate positioning for the operation according to preoperative magnetic resonance imaging (MRI), the positioning accuracy will be reduced when brain drift occurs during the operation. Intraoperative ultrasound imaging combined with preoperative MRI has proved to be reliable, accurate and easy to use, allowing continuous real-time feedback^8^. It is characterized by fast, relatively economical and easy to repeat, and there is no need to interrupt the operation. It is particularly useful when matched with preoperative MRI or CT, because the virtual navigator can provide correct continuous acoustic examination of ultrasound^9^. However, in practice, there are still deficiencies. Firstly, special navigation intraoperative ultrasound equipment is not equipped in most hospitals. Secondly, compared with MRI or CT images, the reading of ultrasound images is extremely difficult for neurosurgeons who lack training.

In summary, the simplicity and availability of equipment and software are the main advantages of 3D Slicer combined Sina/MosoCam multimodal system. The system has many advantages such as simple operation, easy learning, accurate positioning and free. It is considered to be a new technology that is practical, reliable, easy to diagnose and preoperative planning, and is suitable for promotion and use in neurosurgery and other surgical departments of all medical institutions.

## Supplementary Information

Supplementary Legends.

Supplementary Video 1.

## Data Availability

All data generated or analysed during this study are included in this published article and its supplementary information files. 3D slicer free download link: https://slicer.org The old version of Sina has been taken off the shelves, and the latest version can be obtained for free through the following link: https://h5.clewm.net/?url=qr61.cn%2FoWV3W3%2FqnURKgD&hasredirect=1). MosoCam has been removed from the Apple App store at present. Our mobile phones can continue to be used, but those that have not been downloaded before cannot be obtained, but they can be replaced by the re-exposure camera App, which can be obtained free of charge in the Apple App store.
