# Supplementary material for: Clinical application of 3D Slicer combined with Sina/MosoCam multimodal system in preoperative planning of brain lesions surgery
Source: Sci Rep. 2022 Nov 10;12:19258. doi: 10.1038/s41598-022-22549-7 (PMC9649692; doi:10.1038/s41598-022-22549-7)
Supplement: Supplementary file 1 — Supplementary Legends. [file 41598_2022_22549_MOESM1_ESM.docx]

Video 1. Preoperative projection positioning was performed using Apple mobile phone APP MosoCam
